# A Manual of Surgical Treatment

**Published:** 1902-10

**Authors:** 


A MANUAL OF SURGICAL TREATMENT. By W. WATSON CHEYNE, M. B., F. R. C. S., F. R. S., Professor of Surgery in King's College, London; Surgeon to King's College Hospital, etc.; and F. F. BURGHARD, M. D. and M. S. (Lond.), F. R. C. S., Teacher of Practical Surgery in King's College, London; Surgeon to King's College Hospital, etc. In seven imperial 8vo volumes, with illustrations. Volume VI., 498 pages with 124 illustrations. Philadelphia and New York: Lea Brothers & Co. 1902. [Price, cloth, $5.00 net.]

This was to have been the last of this very excellent work, but the authors announce that they have been compelled to make the volume into two sections, the first of which has now been issued, the second to follow "in a few months." The reason for this is that to have crowded all the material into one volume would have made too bulky a book and would have, too, delayed publication much longer. As in previous volumes, which have been reviewed at length, the illustrations are worthy of especial attention. They are admirably made and splendidly presented; in such a manner as to really be of some use other than as mere pictures to make good the page in the index which is given up to "illustrations."

In this volume the surgical affections of the tongue and floor of the mouth are considered at length and in the same clear cut style and with the same careful attention to detail and explanation which has characterised the previous literary work of the joint authors. Then follows in regular order the surgical affections of the pharynx, esophagus and neck; the surgical affections of the abdomen. This last section is given most careful consideration and detailed description. The division includes affections of the abdominal wall; methods of examination of the stomach; injuries of the stomach and gastrostomy; gastric ulcer; cancer of the stomach; injuries of the omentum, mesentery and intestines; acute intestinal obstruction; intussusception; chronic intestinal obstruction; inflammatory affections of the intestines; peritonitis; hernia; fecal fistula and artificial anus. Much space and many illustrations are given to hernia and appendicitis and the book, on the whole, is well up to the standard which the authors set for themselves when they gave the first volume to the public. N.W.W.

